# The Impact of Treatment Adherence for Patients With Diabetes and Hypertension on Cardiovascular Disease Risk: Protocol for a Retrospective Cohort Study, 2008-2018

**DOI:** 10.2196/13571

**Published:** 2019-05-31

**Authors:** Min Su, Victoria Haldane, Ross Upshur, Frank Sullivan, France Légaré, Michelle Greiver, Xiaolin Wei

**Affiliations:** 1 School of Public Administration Inner Mongolia University Hohhot China; 2 Dalla Lana School of Public Health University of Toronto Toronto, ON Canada; 3 Lunenfeld Tanenbaum Research Institute Sinai Health Systems Toronto, ON Canada; 4 Department of Family and Community Medicine Faculty of Medicine University of Toronto Toronto, ON Canada; 5 School of Medicine University of St Andrews North Haugh United Kingdom; 6 Faculty of Medicine Laval University Quebec, QC Canada; 7 North York General Hospital Toronto, ON Canada

**Keywords:** treatment adherence, cardiovascular disease, primary care

## Abstract

**Background:**

Cardiovascular disease (CVD) is the leading cause of death globally and in Canada. Diabetes and hypertension are major risk factors for CVD events. Despite the increasing availability of effective treatments, the majority of diabetic and hypertensive patients do not have adequate blood pressure and glycemic control. One of the major contributors is poor treatment adherence.

**Objective:**

This study aims to evaluate the impact of treatment adherence for patients with both diabetes and hypertension on acute severe CVD events and intermediate clinical outcomes in Canadian primary care settings.

**Methods:**

We will conduct a population-based retrospective cohort study of patients living with both diabetes and hypertension in Ontario, Canada, between January 1, 2008, and March 31, 2018. The Social Cognitive Theory will be used as a conceptual framework by which to frame the reciprocal relationship between treatment adherence, personal factors, and environmental determinants and how this interplay impacts CVD events and clinical outcomes. Data will be derived from the Diabetes Action Canada National Data Repository. A time-varying Cox proportional hazards model will be used to estimate the impacts of treatment adherence on CVD morbidity and mortality. Multivariable linear regression models and hierarchical regression models will be used to estimate the associations between treatment adherence of different medication categories and intermediate clinical outcomes. Our primary outcome is the association between treatment adherence and the risk of acute severe CVD events, including CVD mortality. The secondary outcome is the association between treatment adherence and intermediate clinical outcomes including diastolic and systolic blood pressures, glycated hemoglobin, low-density lipoprotein cholesterol, and total cholesterol. Owing to data limitation, we use medication prescriptions as a proxy to estimate treatment adherence. We assume that a patient adhered to medications if she or he had any prescription record in the 4 preceding quarters and 1 quarter after each quarter of interest. Acute severe CVD events are defined based on the World Health Organization’s Monitoring Trends and Determinants in Cardiovascular Disease Project, including acute coronary heart disease, stroke, and heart failure. As causes of death are not available, the number of CVD deaths will be computed using the most recent systolic blood pressure distributions and the population attributable risks related to systolic blood pressure level.

**Results:**

The project was funded by Diabetes Action Canada (reference number: 503854) and approved by the University of Toronto Research Ethics Board (reference number: 36065). The project started in June 2018 and is expected to be finished by September 2019.

**Conclusions:**

The findings will be helpful in identifying the challenges of treatment adherence for diabetic and hypertensive patients in primary care settings. This will also help to develop intervention strategies to promote treatment adherence for patients with multi-morbidities.

**International Registered Report Identifier (IRRID):**

DERR1-10.2196/13571

## Introduction

### Background

Cardiovascular disease (CVD) is the leading cause of death in Canada, accounting for one-third of deaths nationally [1]. Diabetes and hypertension are major risk factors for CVDs [2-4]. In 2017, approximately 7.3% and 17.8% of Canadians aged 12 years and older reported being diagnosed with diabetes and hypertension, respectively [5]. Diabetes and hypertension are the 2 most common comorbid chronic diseases seen in primary care consultations. Hypertension is reported in over two-thirds of patients with type 2 diabetes [6], whereas nearly 50% of patients with hypertension are diabetic [7]. Despite the increasing availability of effective treatment regiments and guidelines, approximately half of the treated patients do not have adequate blood pressure and glycemic control [8,9]. One of the major contributors to inadequate control is poor treatment adherence [9,10]. Treatment adherence is defined as the degree to which the patient’s behavior corresponds with the agreed recommendations from a health care provider [11]. Adherence to antihypertension and antidiabetes medications is proven to reduce CVD morbidity and mortality, hospitalizations, and health expenditure [11-17]. Given the potential impacts of treatment adherence on CVD morbidity and mortality, quantifying treatment adherence and its impacts will help in the development of intervention strategies to improve treatment adherence for patients in primary care settings. This includes the use of patient-centered approaches such as concordance, where doctors elicit patients’ views, inform patients of the pros and cons of taking medicine, and involve patients in treatment decision making [18]. This type of informed and shared decision making is believed to improve patient satisfaction, adherence, and treatment outcomes [19]. Unfortunately, the process of patient-doctor communication is often not recorded and cannot be quantified using traditional medical records to understand adherence behaviors.

Treatment adherence can be measured using either subjective or objective methods. One of the most frequently used subjective measures is the Morisky Medication Adherence Scale [20], which is a patient self-reported tool with 8 items related to medication-taking behaviors that can be transformed into an adherence score [21,22]. Morisky scale heavily relies on patient’s attitudes toward their medications rather than actual medication-taking behaviors and is vulnerable to significant recall bias [21,22]. Patients tend to underreport their nonadherence to avoid disapproval from their physicians or researchers administering the test [17]. A major limitation of conducting objective measures of adherence is that administrative databases often do not record medication taking. Proxy measures such as prescription refills are used [17] with the assumption that prescription refill patterns correspond to medication-taking behavior [23-25]. However, medication refill patterns can vary substantially across different health care providers and settings.

Up to now, there were limited studies investigating medication treatment adherence and its association with CVD events [26-35] and clinical outcomes [35-40] (Multimedia Appendix 1). Using refill adherence, previous studies have reported that lower adherence (<80%) levels were associated with higher risk of CVD [26], all-cause mortality, and hospitalization for CVD after adjusting for demographic, socioeconomic status (SES), and baseline clinical characteristics [29]; higher adherence to statins (>80%) was associated with significant reduction in low-density lipoprotein cholesterol (LDL-C) in patients with diabetes [36]. In these studies, refill adherence was measured by the medication possession ratio (MPR), reporting the proportion of days with medications on hand during the follow-up. In Canada, there have been 12 studies that were investigated for treatment adherence of patients with diabetes [41-44], hypertension [30-32,34,45,46], or both [47] (Multimedia Appendix 1). Of these studies, 5 reported a negative association between treatment adherence and chronic heart failure [30,31], end-stage renal disease [31], mortality [32], a composite of all-cause death and hospitalization for acute myocardial infarction, heart failure, or stroke [33], and combined CVD events (coronary artery disease, cerebrovascular disease, and chronic heart failure) and hospitalization costs [34]. Using refill adherence, previous studies have showed that lower treatment adherence (<80%) was associated with higher risk of coronary disease [36], cerebrovascular disease [36], and chronic heart failure [36], after adjustment for demographic and SES [30]. These studies had several common limitations. First, few studies examined treatment adherence among patients with multimorbidities, such as both diabetes and hypertension, which accounted for the majority of these patients and is in line with studies showing that multimorbidity and medical complexity increase with age [48]. Second, most studies examined only one type of medication adherence, not considering the combined benefits of adhering to multiple medications in preventing CVD events and mortality [49,50]. Third, many studies were cross-sectional in design and relied on survey- or hospital-based electronic medical records (EMRs) or had a relatively shorter follow-up time (<10 years), which limited the studies’ ability to inform clinical practice at the primary care level.

### Theoretical Framework

**Figure 1:**
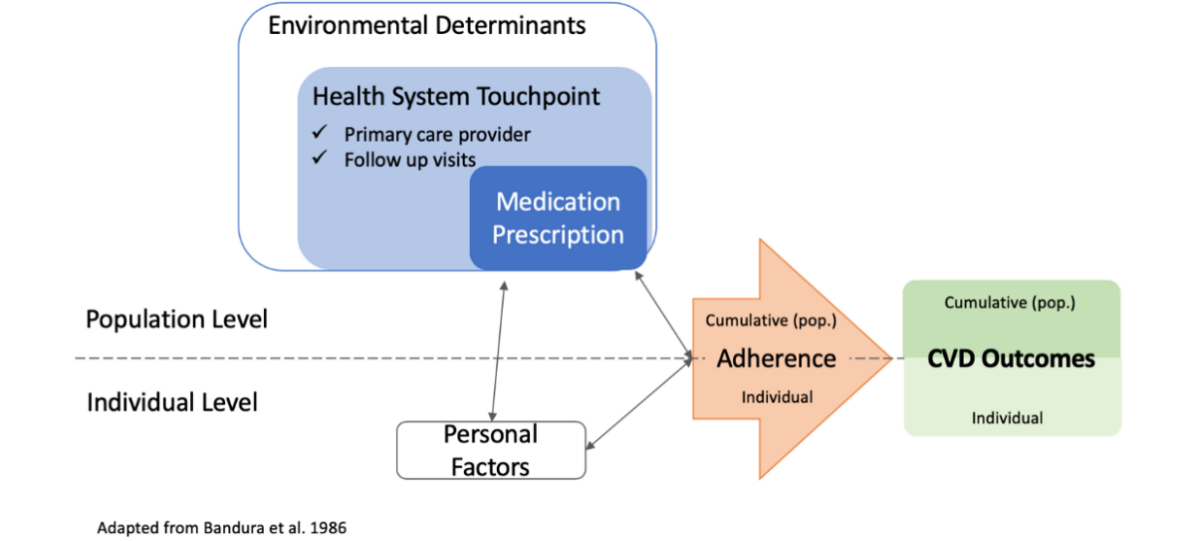
Theoretical framework. CVD: cardiovascular disease.

Treatment adherence is largely viewed, and measured, as a behavior at the individual level [51]. However, adherence is multifactorial and influenced by a host of environmental determinants [52-54]. Although often neglected in adherence studies, environmental determinants have been reported as barriers to adherence at the individual level [55]. Thus, we intend to use Social Cognitive Theory as a lens through which to conceptualize both personal-related factors and environmental determinants that may influence treatment adherence. Social Cognitive Theory explains a reciprocal relationship between that behavior, personal factors, and environmental determinants, and in the case of health, this interplay impacts health outcomes (Figure 1) [56,57].

Other studies of adherence at the individual level have employed the Health Belief Model or Theory of Planned Behavior to understand how individuals may engage in adherence behavior; however, these frameworks are limited in application at the population level and do not adequately explicate environmental determinants of adherence behaviors [58-60]. Similarly, theoretical frameworks that include environmental determinants at the population level, such as the Social-Ecological Model, while useful in revealing the environmental, organizational, and social factors influencing health, are limited in ability to highlight the behavioral mechanisms underpinning adherence [61]. Thus, the Social Cognitive Theory, which explicitly includes environmental determinants and personal factors as contributing to behavior, is well placed to investigate the population factors that impact adherence.

Our study seeks to examine the ways in which medication prescription, as proxy for adherence, impacts on CVD events and clinical outcomes. As shown in our conceptual framework (Figure 1), the behavior of treatment adherence operates not only at the individual level but also cumulatively at the population level. Similarly, health system touchpoints exist and exert impact at the population level as part of the environmental determinants of treatment adherence. These factors also interact with and are influenced by personal factors; however, in our analysis, we are controlling for these personal-level elements to highlight the role of medication prescription as an environmental determinant. We hypothesize that these environmental factors, measured by primary care provider characteristics, medication prescription, and medication regimen complexity over time, in turn, would impact on individual CVD events and clinical outcomes. Ultimately, this lens allows us to consider how population-level considerations, such as health system touchpoints, impact adherence at the population level. The results will provide evidence that may inform health policy and specific health service interventions.

### Objectives and Hypotheses

Objective 1: The first objective was to assess the impacts of treatment adherence on acute severe CVD events in Ontario between January 1, 2008, and March 31, 2018 (10 years).

Hypothesis 1: Patients with a lower adherence rate of one or more medications (antihypertension, antidiabetes, statins, and aspirin) are more likely to develop acute severe CVD events (including death from CVD), adjusting for potential confounding factors.

Objective 2: The second objective was to assess the impacts of treatment adherence on intermediate clinical outcomes in Ontario between January 1, 2008, and March 31, 2018.

Hypothesis 2: Patients with a higher adherence rate are associated with significant improvements in clinical outcomes including diastolic blood pressure, systolic blood pressure, glycated hemoglobin (HbA_1c_), LDL-C, and total cholesterol (TC), adjusting for potential confounding factors.

## Methods

### Study Design and Participants

This is a retrospective cohort study using primary care EMR data. A cohort of patients who were medically diagnosed with both diabetes and hypertension between January 1, 2008, and March 31, 2018, will be included in this study.

### Inclusion and Exclusion Criteria for Participants

#### Time to Enter the Cohort

**Figure 2:**
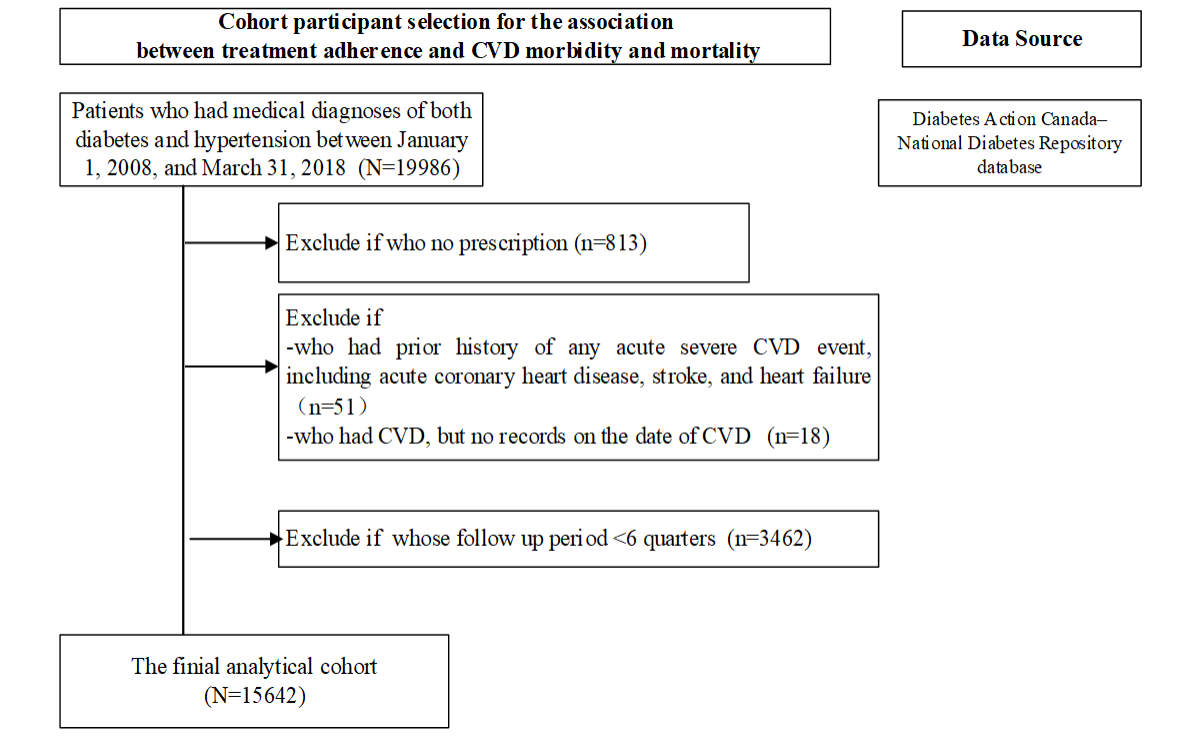
Cohort participant selection for the association between treatment adherence and cardiovascular disease (CVD) morbidity and mortality.

**Figure 3:**
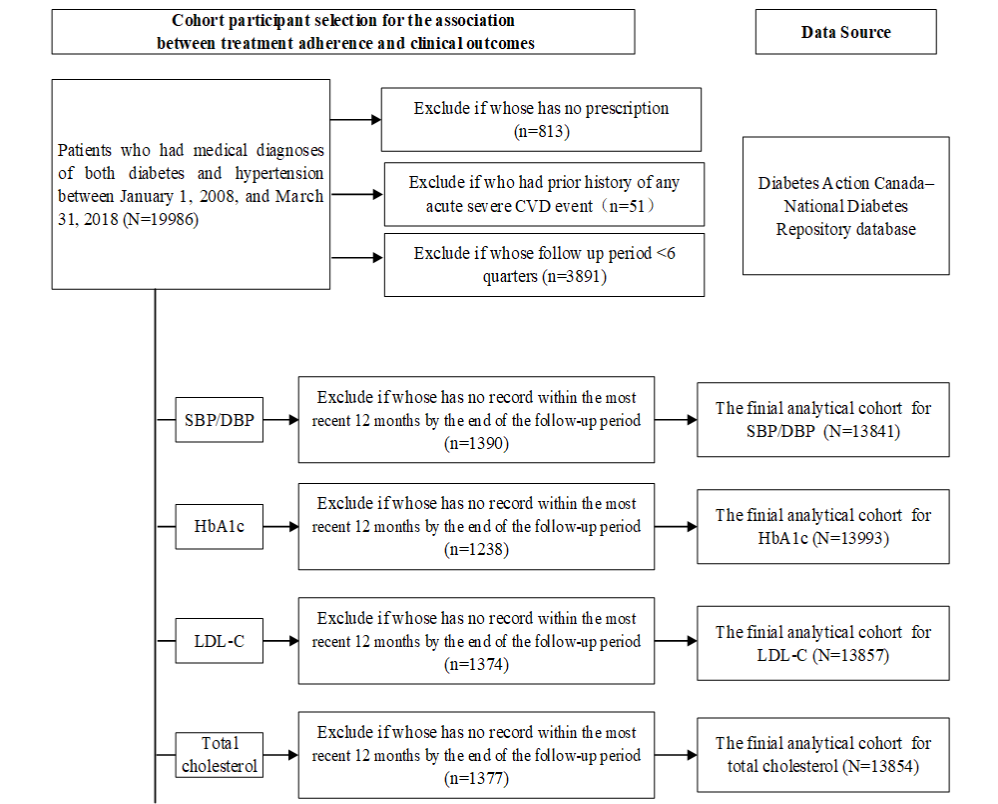
Cohort participant selection for the association between treatment adherence and clinical outcomes. CVD: cardiovascular disease; SBP:systolic blood pressure; DBP: diastolic blood pressure; HbA_1c_: glycated hemoglobin; LDL-C: low-density lipoprotein cholesterol.

Cases entered the cohort when a medical diagnosis of both diabetes and hypertension was present and when a prescription for any antihypertensives or antidiabetic medication was provided in the EMR. The exclusion criteria included (1) patients with a past history of any acute severe CVD event, (2) patients who developed CVD events during follow-up where no date of CVD event was present, and (3) patients whose follow-up period was below 6 quarters as we cannot estimate its treatment adherence. A total of 15,642 eligible participants are identified in the finial study population (Figures 2 and 3).

#### Follow-Up

We will retrospectively follow-up all eligible participants until March 31, 2018 (by months). Follow-up ends when a participant dies, has any acute severe CVD event, or by the end of the study (March 31, 2018). For hypotheses related with clinical outcome, follow-up ends with the participant’s latest diastolic blood pressure, systolic blood pressure, HbA_1c_, LDL-C, and TC outcomes. The follow-up period will be measured approximately in months and treated as an independent variable.

### Data Source

We will utilize the Diabetes Action Canadian National Diabetes Repository as the data source. The Repository contains deidentified data from over 100,000 patients living with diabetes, currently from 4 Canadian provinces (Ontario, Manitoba, Quebec, and Alberta). Data are extracted from primary care EMRs of consenting family physicians and nurse practitioners by regional Practice Based Research Networks who are members of the Canadian Primary Care Sentinel Surveillance Network (CPCSSN) and are managed using previously described processes developed through CPCSSN [61]. The Repository provides a Secure Analytic Virtual Environment, which is a privacy compliant research platform in a high-performance computing center. Approved researchers access the Secure Analytic Virtual Environment remotely to analyze datasets derived from Repository data. All projects are reviewed by the Repository’s Research Governing Committee, composed of at least 50% patients, to ensure the project’s values are consistent with those of patients living with diabetes and of their caregivers. This project was reviewed and approved by the Research Governing Committee.

Deidentified patient data from contributing practices in the Diabetes Repository include the following: (1) patient demographic characteristics, (2) patient health conditions, (3) physical and laboratory examinations, (4) medication prescriptions, (5) risk factors, and (6) comorbidities. A data dictionary that provides information on data elements is available at the Diabetes Action Canada website.

### Exposure and Study Outcomes

#### Treatment Adherence Rate

Medication adherence rate is considered the exposure of interest. Medications will be classified as (1) antidiabetic medications, including metformin, sulfonylurea, and insulin, inhibitors of dipeptidyl peptidase 4, meglitinide, sodium-glucose cotransporter-2 inhibitors, thiazolidinedione, and alpha-glucosidase enzymes, (2) antihypertensive medications, including angiotensin converting enzyme inhibitors, thiazide diuretics, beta-blockers, calcium channel blockers, and angiotensin II receptor blockers, (3) statins, and (4) aspirin. We will measure patient adherence to each type of prescription if prescribed. Theoretically, the adherence rate could be calculated based on the prescription date and refills. However, we cannot depend on the refills as this information varies highly among primary care physicians (eg, some refilled every 3 months, others provided multiple repeats) [62]. Previous research indicated that lag-lead approach is feasible to estimate adherence based on time-dependent associations between different variables (or the same variable) in longitudinal data analysis [63]. We will use this approach to account for the variation in refills. Based on a study using the CPCSSN database, we assume that patients adhered to medications if they had any prescription record in the 4 preceding quarters and 1 quarter after each quarter of interest (lag4, lead1) [64].

#### Cardiovascular Disease Morbidity and Mortality

The primary outcome is the risk of any acute severe CVD events including mortality to identify cohort members who developed acute severe CVD events or who died from acute severe CVD events during the follow-up. Acute severe CVD events are defined based on the World Health Organization’s Monitoring Trends and Determinants in Cardiovascular Disease Project, including acute coronary heart disease (ICD-9 code 410-412, 414), stroke (ICD-9 code 430-438), and heart failure (ICD-9 code 428) [26,65,66].

**Figure 4:**
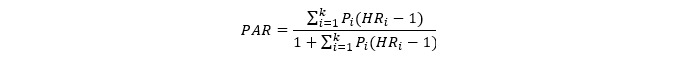
Formula for population attributable risks (PARs).

We could not access the cause of mortality among patients in this cohort owing to data limitation. Thus, we estimate the cause of mortality based on previous research by Bundy et al who offer a method to estimate the association between systolic blood pressure and CVD mortality [67]. We will apply this method to identify cohort members who died from CVD events during the follow-up. This method assumes that the number of CVD deaths could be increased if the population developed higher systolic blood pressure treatment levels. The number of CVD deaths will be computed using systolic blood pressure distributions and the population attributable risks (PARs) related to systolic blood pressure level. A given PAR represents the proportion of CVD deaths that could be increased by higher systolic blood pressure levels. We will divide the most recent systolic blood pressure level into 8 categories (<130, 130-134, 135-139, 140-144, 145-149, 150-154, 155-159, and ≥160 mmHg). The PARs will be calculated using the formula given in Figure 4, where p_i_ is the proportion of the systolic blood pressure category i, HR_i_ is the hazard ratio of CVD deaths in the systolic blood pressure category i, and k is the total number of systolic blood pressure categories. To estimate hazard ratios for CVD mortality comparing each of the 8 systolic blood pressure categories, Bundy et al conducted a network meta-analysis of 42 antihypertensive clinical trials [68]. We will use the hazard ratio of CVD death from the meta-analysis study conducted by Bundy et al. For patients who died during the follow-up period, we assume that the patient died from CVD events if the PAR was higher than 50%.

#### Clinical Outcomes

The secondary outcomes are the most recent clinical treatment outcomes, including diastolic blood pressure, systolic blood pressure, HbA_1c_, LDL-C, and TC.

### Baseline Covariates and Other Covariates

Based on data availability, we will include the following personal-related factors: (1) patients’ demographic and socioeconomic characteristics (ie, sex, age, body mass index, SES, and rurality), (2) risk factors (ie, smoking history, and alcohol history), (3) comorbidities and its duration by the end of the follow-up period (ie, chronic obstructive pulmonary disease, depression, dementia, and Parkinson), and (4) clinical outcomes at baseline (ie, diastolic blood pressure, systolic blood pressure, HbA_1c_, LDL-C, and TC). Environmental determinants contributing to treatment adherence include those related to primary care physicians and health systems: (1) physicians’ demographic characteristics (ie, sex, age, and location type) and (2) complexity of prescription (ie, the types of medication). All covariates will be treated as baseline covariates except that we will measure the incidence of comorbidities and the time to follow-up.

SES is defined according to the Canadian Material Deprivation Index [69]. The Canadian Material Deprivation Index, a proxy for individual-level SES based on the most recent 6-digit residential postal code, is calculated by the average income, percentage without high school graduation, and the employment ratio [70]. SES will be categorized into high, average, and low SES groups.

### Statistical Analysis

The analyses will be described separately for objective 1 and objective 2. We will include participants with nonmissing information on treatment adherence. Multiple imputations will be used to replace missing data for baseline covariates, SES, and comorbidities. Finally, a practice site will be used as a random effect in each model. All analyses will be performed in Stata version 13.0 (Stata Corp LP).

#### Objective 1

For hypothesis 1, a time-varying Cox proportional hazards model will be performed to evaluate the hazard ratio between treatment adherence and the incidence of acute severe CVD and mortality, adjusting for all potential covariates. In addition, the interactions of adherence to multiple medications will be included as a block [71] in the Cox proportional hazards model to examine the combined benefits of adherence in preventing CVD events and mortality (model 1). Treatment adherence and acute severe CVD risk is considered as the exposure of interest and outcomes, respectively. We will measure follow-up time (in months) from the date of entry into the cohort until the date of incident acute severe CVD events, died, or the end of the follow-up period. Kaplan-Meier survival curves for any acute severe CVD and mortality by treatment adherence level will be generated by pooling the survival estimates.

#### Objective 2

For hypothesis 2, 4 multivariable linear regression models will be used to test the association between treatment adherence and the most recent clinical outcomes after adjusting for all potential covariates (model 2-model 5). An adjusted risk ratio with 95% CI will be generated. We will build the hierarchical regression [71] into the linear regression models by including the adherence to different type of medications as a block to test the aggregated contribution of adherence to clinical outcomes, including diastolic blood pressure, systolic blood pressure, HbA_1c_, LDL-C, and TC.

#### Sensitivity Analysis

Previous evidence has also indicated that there is no single correct lag and lead for estimating time-dependent association [63]. Lag and lead choices are a significantly important issue in the generalizability of results [62,72]. Therefore, we will conduct a sensitivity analysis using lag1 and lead 4 (Multimedia Appendix 2). Additionally, we will examine the consistency of the results regarding the following: (1) at different treatment adherence categories (ie, ≥80%, 60% to 80%, 40% to 60%, and ≤40%) and (2) with and without multiple imputation of missing data on all covariates.

### Ethics Approval

The study was approved by the University of Toronto Research Ethics Board (reference number: 36065). Data were deidentified when analyzed.

## Results

The project was funded in July 2017 under Diabetes Action Canada (reference number: 503854). The study was approved by the University of Toronto Research Ethics Board (reference number: 36065). The project was started in June 2018. The results are expected to be finished by September 2019.

## Discussion

### Strengths

CVD events represent a heavy disease burden on individuals and their families, the health system, and society in general. Improving treatment adherence to antihypertensive and antidiabetic medication has been well documented as an effective strategy to prevent CVD events. Compared with previous research, our study has several strengths. First, we will contribute new knowledge on the association between treatment adherence, acute CVD events, and clinical outcomes among patients with both diabetes and hypertension in the primary care setting. Second, we will examine the combined benefits of adhering to multiple medications in preventing CVD events and mortality and clinical treatment outcomes. Finally, our study provides further knowledge by addressing the limitations of previous studies, such as inclusion of important potential confounders such as comorbidities, their duration, and follow-up. For example, previous research reported that mental health conditions (ie, depression, anxiety, and dementia) were important factors when analyzing treatment adherence and CVD events but lacked the ability to identify such mental conditions [73,74]. Many studies have used the Charlson Comorbidity Index [27-29] to explore the combined effects of comorbidities (ie, chronic obstructive pulmonary disease, depression, chronic kidney disease, and dementia), but this approach does not reflect the role of individual comorbidities. Furthermore, these studies did not control for duration of comorbidities. Finally, baseline clinical characteristics such as HbA_1c_, lipids, blood pressure, and body mass index are also important confounding factors, which may have a direct impact on CVD risk and clinical treatment outcomes [75]. However, few studies control for these factors. We will address the limitations by controlling body mass index, diastolic blood pressure, systolic blood pressure, HbA_1c_, LDL-C, and TC. Owing to the data availability, the percentage of missing data at baseline is as follows: body mass index (25.25%), diastolic blood pressure (23.54%), systolic blood pressure (18.27%), HbA_1c_ (28.09%), LDL-C (46.21%), and TC (45.92%). We will use multiple imputation and consequently carry out a sensitivity analysis with and without imputation of missing data on all covariates. Our study will be the first population-based cohort study that systematically investigates the impacts of treatment adherence for patients with both diabetes and hypertension on CVD morbidity and mortality, and clinical treatment outcomes using a longitudinal and large-scale primary care EMR data. Our findings will help to identify challenges in treatment adherence for patients with diabetes and hypertension in primary care settings. Through this study, we hope to provide valuable evidence for policy and practice to inform the design and implementation of primary care health services to support adherence among patients living with diabetes and hypertension.

### Limitations

Several limitations should be noted in our study. First, this study is a retrospective study. All data were recorded from routine EMRs with possible errors and omissions. Thus, CVD may not be captured in full. As recording the medical diagnosis of diabetes, hypertension, and CVD is the responsibility of primary care physicians, there may be delays in EMR input. Second, treatment adherence is measured using a proxy, not a real measure. Theoretically, patients with both diabetes and hypertension are regarded as having a high risk of CVD events and should take prescribed medications consistently. We assume that prescription patterns correspond to medication-taking behavior. Third, owing to data limitations, we estimate CVD mortality using systolic blood pressure distributions and the population attributable risks related to the systolic blood pressure level. Fourth, aspirin is an over-the-counter medication that patients can obtain from pharmacies without a prescription. Thus, we may underestimate the adherence rates for aspirin. Finally, we employed the Social Cognitive Theory to explore the reciprocal relationship between that treatment adherence and diabetes/hypertension management outcomes (such as CVD), in the context of personal factors and environmental determinants. However, there were many factors not recorded in our database. For example, there were no variables such as primary language, ethnicity, health literacy, employment status, and marital status, which are factors contributing to patient understanding of the treatment or related to their daily management. There were no process variables recorded such as patient’s level of involvement in the treatment decision-making process, understanding of their disease, and family and social support. In addition, there was a lack of reporting on physician-specific variables, such as the level of communication to patients on the benefits and adverse effects of a prescription nor did we have variables related to the health system such as access to primary care and primary care models [15]. These limitations may lead to potential bias.

## Appendix

Multimedia Appendix 1 Study characteristics.

Multimedia Appendix 2 Lag lead method.

## Abbreviations

CPCSSN: Canadian Primary Care Sentinel Surveillance Network
CVD: cardiovascular disease
EMR: electronic medical record
HbA_1c_: glycated hemoglobin
LDL-C: low-density lipoprotein cholesterol
PAR: population attributable risk
SES: socioeconomic status
TC: total cholesterol
