# Supplementary material for: The Impact of Treatment Adherence for Patients With Diabetes and Hypertension on Cardiovascular Disease Risk: Protocol for a Retrospective Cohort Study, 2008-2018
Source: JMIR Res Protoc. 2019 May 31;8(5):e13571. doi: 10.2196/13571 (PMC6658229; doi:10.2196/13571)
Supplement: Multimedia Appendix 1 [file resprot_v8i5e13571_app1.pdf]

Multimedia Appendix 1: [supplementary table]

Supplementary table: study characteristics

Study	Study design	Location	Population	Sample size	Follow up years	Measurement	Medication Class	Outcome	Confounders	Methods	Findings
Karlsson et al.2017 [26]	Retrospective cohort study	Sweden	Patients with Type 2 diabetes aged ⩾18 years old	86,568	4	Medication possession ratio (refill adherence)	Lipid-lowering medications (insulin only, other glucose-lowering medication only, insulin and other glucose-lowering medication)	Medication adherence and its impact on CVD events (myocardial infarction, ischemic heart disease, stroke and unstable angina)	Baseline socioeconomic status, comorbidities, clinical characteristics (diabetes duration, HbA1c, estimated glomerular filtration rate, BMI, blood lipid levels, blood pressure, microalbuminuria, macroalbuminuria) physical activity and smoking	Cox proportional hazards regression, Kaplan-Meier survival curves	The hazard ratios for CVD ranged 1.33–2.36 in primary prevention patients and 1.19–1.58 in secondary prevention patients, for those with MPR ⩽80% (p<0.0001)
Kim et al.2018 [27]	Retrospective cohort study	South Korea	Type 2 diabetes aged ⩾40 years old	28,158	10	Medication possession ratio (refill adherence)	Oral hypoglycemic agents (biguanide, sulfonylurea, and others)	Medication adherence and its impact on the long-term all-cause mortality and hospitalization for CVD events (cerebrovascular disease, myocardial infarction)	Baseline age, sex, disability, insurance type, monthly contributions, medical institution type, Charlson Comorbidity Index, hypertension, active ingredient of oral hypoglycemic agent (biguanide, sulfonylurea, and others)	Cox-proportional hazard regression	The hazard ratio for all-cause-mortality of the lowest adherence group (<0.20) was 1.45 as compared to the highest adherence (⩾0.8). The HR for all-cause-mortality associated with adherence levels of 0.60–0.79, 0.40–0.59, and 0.20–0.39 were 1.19, 1.26, and 1.34, respectively (P< 0.001). Diabetic patients with the lowest adherence level had elevated risk

										for CVD (HR = 1.41, P< 0.001)
Shin et al.2013 [28]	Retrospective cohort study	South Korea	Hypertensive patients aged ⩾18 years old	40,408	4	Medication possession ratio (refill adherence)	Antihypertensive agents (calcium channel blockers, diuretics, ACEI, ARB, beta-blockers)	Medication adherence and its impacts on CVD events (ischemic heart disease, stroke, chronic heart failure) and hospitalization	Baseline age, gender, insurance type, cardiovascular risk at baseline, diabetes, dyslipidemia, Charlson Comorbidity Index, number of classes of medications given upon initial prescription and previous hospitalizations	Cox proportional hazards regression	Nonadherence increased the risk of all adverse health outcomes, including all-cause mortality and hospitalization for CVD (hazard ratio: 1.57)
Yang et al.2017 [29]	Retrospective cohort study	Atlanta, USA	Patients with hypertension; Medicare fee-for-service beneficiaries aged 66 to 79 years	155,597	7	Medication possession ratio (refill adherence)	Antihypertensive agents (calcium channel blockers, diuretics, ACEI, ARB, beta-blockers)	Medication adherence and its impact on CVD events (acute myocardial infarction, ischemic heart disease, stroke/transient ischemic attack, and heart failure)	Baseline age, gender, race, urban and rural residence, status of low-income subsidy, statin use, number of classes of antihypertensives on hand during follow-up, Charlson Comorbidity Index, out-of-pocket antihypertensive expenditure per day during follow-up	Cox proportional hazards regression	1) Around 60% were classified as being highly adherent to their medication (access to medication on ⩾80% of days);<br>2) There appeared to be a threshold effect in reducing CVD events at around PDC 80%, above which the risk for CVD reduced substantially
Perreault et al.2009 [30]	Case–control study	Quebec, Canada	Hypertensive patients aged 45 to 85 years old	82,320	6	Medication possession ratio (refill adherence)	Antihypertensive agents (calcium channel blockers, diuretics, ACEI, ARB, beta-blockers)	Medication adherence and its impact on chronic heart failure	Developing coronary artery disease, a cerebrovascular disease, a peripheral arterial disease or any other cardiovascular events, diabetes, dyslipidemia,	Cox proportional hazards regression	High adherence level (95%) to AH therapy compared with lower adherence level (60%) was associated with an additional reduction of CHF events (RR: 0.89). Risk factors for CHF were being on social assistance, diabetes,

									comorbidity index score during follow up		dyslipidemia, higher chronic disease score and developing a cardiovascular condition during follow-up
Roy et al.2013 [31]	Retrospective cohort study	Quebec, Canada	Hypertensive patients aged 45 to 85 years old	185,476	8	Medication possession ratio (refill adherence)	Antihypertensive agents	Medication adherence and its impact on end-stage renal disease	Age, gender, social assistance, diabetes, dyslipidemia, ischemic heart disease, cerebrovascular disease, peripheral arterial disease, chronic heart failure, infectious disease, chronic disease score, use of antihypertensive agents in the year before event, nephrotoxic drugs	Cox proportional hazards regression	A high adherence level of 80% or more to antihypertensive agent(s) compared to a lower one was related to a risk reduction of ESRD (hazard ratio 0.67)
Liu al.2014 [32]	Retrospective cohort study	British Columbia, Canada	Patients with hypertension	148,914	14	Medication possession ratio (refill adherence)	Antihypertensive agents (calcium channel blockers, diuretics, ACEI, ARB, beta-blockers)	Medicine adherence and its impact on mortality according to ethnicity (All-cause death)	Age, gender, Charlson Comorbidity Index, socioeconomic status, urban and rural residence	Multivariable logistic regression models	1) South Asian and Chinese patients with hypertension are less likely to adhere to antihypertensive medications in Canada. 2) High adherence to antihypertensive medications was associated with reduced mortality in Caucasian, but the association was not observed in South Asian and Chinese patients
Verma et al.2018[33]	Retrospective cohort study	Ontario, Canada	Hypertensive patient aged 66 years old or over	13,350	5	Medication possession ratio (refill adherence)	ACEI, ARB, plus one thiazide diuretic	Medicine adherence and its impact on all-cause death and hospitalization for	Baseline age, gender, neighborhood income quintile,	Propensity score matching, Cox	1) The time to the first instance of discontinuation: 150 days for multipill

								acute myocardial infraction, heart failure, or stroke	urban and rural residence, Charlson Comorbidity Index, health care utilization history, index medication dose	proportional hazards regression	combinations vs 191 days for single-pill fixed-dose combinations; 2) The proportion of total days covered: 42% for multipill combinations vs 70% for single-pill fixed-dose combinations
Dragomir et al.2010 [34]	Retrospective cohort study	Quebec, Canada	Hypertensive patients aged between 45 and 60	59,647	3	Medication possession ratio (refill adherence)	Antihypertensive agents (calcium channel blockers, diuretics, ACEI, ARB, beta-blockers)	Medication adherence and its impact on CVD events (coronary artery disease, cerebrovascular disease, and chronic heart failure) and hospitalization costs	Age, gender, social assistance status, severity of hypertension, comorbidities (diabetes), hospitalization history, mean number of physician consultations per year	Polytomous logistic analysis, multivariate logistic regression	Low adherence to AH agents was correlated with a higher risk of vascular events, hospitalization, and greater healthcare costs
Wu et al. 2010 [35]	Retrospective cohort study	Taiwan	Hypertensive patients	29,685	1	Medication possession ratio (refill adherence)	Antihypertensive agents (calcium channel blockers, diuretics, ACEI, ARB, beta-blockers)	Medication adherence and its association with blood pressure (BP) control, cardiovascular disease (ischemic heart disease, stroke, and other diseases of the circulatory system) hospitalization, and all-cause hospitalization	Age, sex, smoking, drinking history, BMI, hypertension history, Charlson Comorbidity Index, and baseline BP	Multiple logistic regression models	Poor medication adherence (<80%) was associated with poor BP control (odds ratio (OR) = 1.20), CVD hospitalization (OR = 1.43), and all-cause hospitalization (OR = 1.47)
Raebel et al.2017 [36]	Retrospective cohort study	United States	Type 2 diabetes aged ⩾65 years old	129,040	2	Medication possession ratio (refill adherence)	ACEI, ARB, statin	Medication adherence and its impacts on clinical BP, LDL-C	Gender, race/ethnicity, whether the patient was enrolled in the health plan the full	Poisson regression	Compared to no comorbidity, high comorbidity (⩾ 4) was associated with lower ACEI/ARB (risk ratio 0.88 or statin (0.91)

								calendar year, region, , income and education, the number of medication classes the patient was taking, prescription co-payment, out-of-pocket		adherence. ACEI/ARB adherence was not associated with blood pressure < 140/90 mm Hg in patients ⩾ 85 years (RR 1.01) or with multiple comorbidities (e.g., 3: RR 1.04). Statin adherence and LDL-C < 100 mg/dl were associated in all elderly age groups (e.g., ⩾ 85: RR 1.13) and comorbidity levels (e.g., ⩾ 4: RR 1.13)
Frias et al.2017 [37]	Prospective, open-label, cluster-randomized pilot clinical trial	California and Colorado, United States	Adults with uncontrolled hypertension and type 2 diabetes	109	12 weeks	Medication taking behavior by a digital medicine offering (DMO)	Antihypertensive agents (calcium channel blockers, diuretics, ACEI, ARB), antidiabetic agents (biguanide, sulfonylurea), and stain	Effectiveness of Digital Medicines (adherence) to improve clinical outcomes (BP, HbA1c, and LDL-C)	Age, gender, and race	Mixed-effects regression model	For patients failing hypertension and diabetes oral therapy, this DMO, which provides dose-by-dose feedback on medication ingestion adherence, can help lower BP, HbA1c, and LDL-C, and promote patient engagement and provider decision making
Cohen et al.2017 [38]	Randomized controlled behavioral intervention study	New York	Adults with type 2 diabetes aged 30 years or over	526	1	Medication possession ratio (refill adherence), Morisky Medication-taking Scale	Oral hypoglycemic agent	Medication adherence and its impacts on HbA1c	Sex, age, race/ethnicity, work status, marital status, income, education, and birthplace were collected, BMI, years with diabetes, and insulin use in the previous year	Multivariable logistic regression	Lowest MPR was significantly (p=0.008) associated with highest HbA1c

Krapek et al.2004 [39]	Retrospective cohort study	United Kingdom	Adults with type 2 diabetes aged 18 years or over	301	9	Morisky Medication-taking Scale	Hypoglycemic agent (biguanide, sulfonylurea, insulin, α-glucosidase inhibitors, thiazolidinediones)	Medication adherence and its impacts on HbA1c	Number of antidiabetic agents, insulin versus noninsulin regimen, number of diabetes complications, duration of diabetes, patient age, gender, ethnicity, BMI, educational attainment, clinic site socioeconomic status	Multivariable regression	Good adherence (Morisky score ⩾3) was associated with a 10% lower total HbA1c (p = 0.0003)
Lee et al.2006 [40]	A multiphase, prospective study with an observational phase and a randomized controlled trial	Walter Reed Army Medical Center (Washington DC), United States	Hypertensive patients aged 65 years or older	200	6 months	Actual medication taking behavior	Antihypertensive agents (calcium channel blockers, diuretics, ACEI, ARB, beta-blockers, clonidine, stain)	Medication adherence and its associated effects on BP and LDL-C	Gender, age, race, education, number of comorbidities	Multivariable regression	A pharmacy care program led to increases in medication adherence, medication persistence, and clinically meaningful reductions in BP
Gentil et al.2015[41]	Longitudinal study	Quebec, Canada	Patients aged 45-85 years old or over with diabetes	301	3	Medication possession ratio (refill adherence)	Antihypertensive agents	Medicine adherence and its impact on health care cost	Age, sex, marital status, education, Charlson Comorbidity Index) and OHA exposure in previous year	Generalized linear regression models	1) 74.4% of patients were adherent to the prescribed medication; 2) Nonadherence among people without depression or anxiety was associated with higher total health care costs ($4,48); nonadherence among people with depression or anxiety ($11,12)
Simard et al.2015[42]	Retrospective cohort study	Quebec, Canada	Patients aged 45-85 years old or over with diabetes	160,231	9	Medication possession ratio: the number of days of medication	Antidiabetic agents (sulfonylureas, biguanides,	Medicine adherence	Age, gender, residential status (rural vs. urban), annual drug, copayment ceilings,	Cox proportional hazards regression, Kaplan-Meier	67% of patients were adherent to the prescribed medications

						supply over a given follow-up period	meglitinides, thiazolidinediones, alpha-glucosidase inhibitors, and dipeptidyl peptidase-4 inhibitors)		initial prescribers, number of pharmacologic agents, hospital admissions and physician visits history, comorbidities	survival curves	
Guénette et al.2016[43]	Cross-sectional study	Quebec, Canada	Patients aged 18 years old or over with diabetes	901	NA	8-item morisky medication adherence scale	Insulin	Medicine adherence and its determine factors	Age, sex, main occupation, work schedule, household composition and family income, social support, clinical variables included depressed mood, anxiety, and self-rated mental health, diabetic duration	Parsimonious logistic regression model	1) only 45% expressed high adherence with their non-insulin antidiabetic treatment; 2) factors like age, habit, number of years since a diabetes diagnosis and number of doses prescribed daily are associated with medicine adherence
Grégoire et al.2010 [44]	Retrospective cohort study	Quebec, Canada	New users of antihyperglycemic drug aged 18 years or over	98,940	6	Persistence with initial therapy was defined as continuously refilling a prescription	Antidiabetic agents (metformin, insulin secretagogues sulfonylureas, insulin secretagogues nonsulfonylureas, TZDs, and alpha-glucosidase inhibitor)	Persistence	Age, sex, calendar year of treatment initiation, hospitalization for diabetes, the number of different prescribed medications claimed in the year before treatment initiation, for obesity and mental disorder	Cox proportional hazards regression, Kaplan-Meier survival curves	Compared to diabetic patients initiated on metformin, those initiated on sulphonylureas displayed poorer persistence patterns
Friedman et al.2010[45]	Retrospective cohort study	Ontario, Canada	Patients age 60 years or over with hypertension	207,473	8	Persistence with initial therapy was defined as continuously refilling a prescription	Antihypertensive agents (calcium channel blockers, diuretics,	Persistence	Age, gender, cohort year, drug class, Charlson comorbidity index score	Multivariable logistic regression models	Therapy persistence at 1 year and median time of persistence were 71.5% and 3.07 years, respectively

							ACEI, ARB, beta-blockers, clonidine)				
Gee et al.2012 [46]	Cross-sectional study (self-reported data)	Canada (CCHS)	Hypertensive patients 20 years old or over	6142	NA	Self-reported medication use, starting and stopping medication	Antihypertensive agents	Medication adherence and its determining factors	Gender, age, ethnicity, education, total household income, marital status, and urban or rural residence, BMI, smoking status, presence of diagnosed diabetes or cardiovascular disease, self-rated general and mental health, perceived daily stress, and self-reported physical activity	Log-binomial regression	Compatible with high rates of hypertension control, most Canadians diagnosed with hypertension take medications and report adherence
Natarajan et al.2013 [47]	Cross-sectional study (self-reported data)	Nova Scotia, Canada	Patients with Type 2 diabetes mellitus (DM) and hypertension	527	NA	8-item Morisky medication adherence scale		Medicine adherence and its determining factors	Gender, age, education, duration of hypertension, prescription history, exercise and diet	Multivariate model	1) 77.4% of patients reported high adherence, 20.9% reported medium adherence, and 1.7% reported low adherence; 2) Factors like demographic characteristics, knowledge, beliefs, behavior, health care provider relationships, and health system influence are associated with adherence
