# Supplementary material for: The Impact of Treatment Adherence for Patients With Diabetes and Hypertension on Cardiovascular Disease Risk: Protocol for a Retrospective Cohort Study, 2008-2018
Source: JMIR Res Protoc. 2019 May 31;8(5):e13571. doi: 10.2196/13571 (PMC6658229; doi:10.2196/13571)
Supplement: Multimedia Appendix 2 [file resprot_v8i5e13571_app2.pdf]

Multimedia Appendix 2: [supplementary figure]

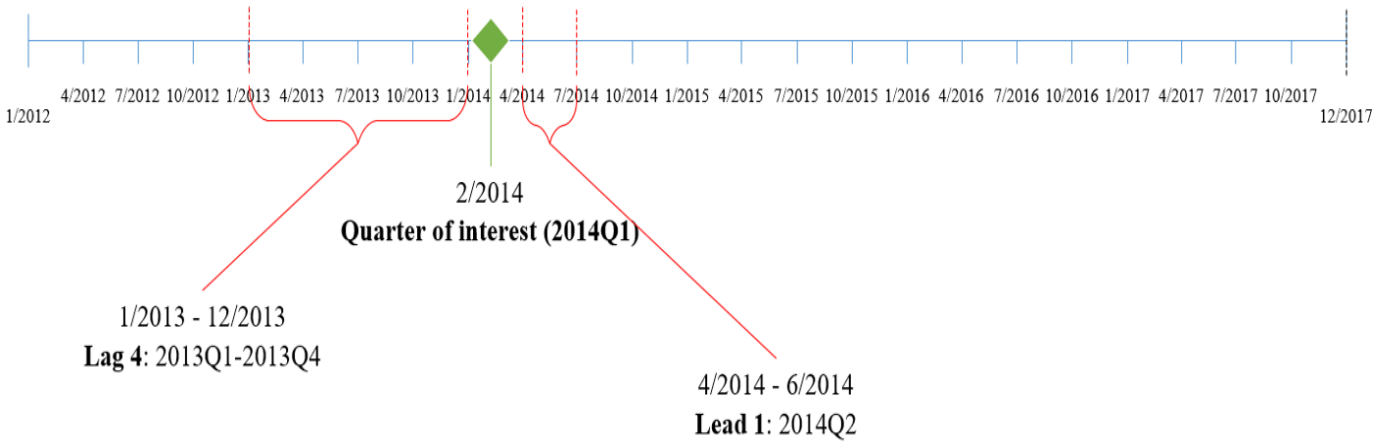

Legend: start follow-up quarter: 2013Q1, the lag 4 of 2013Q1 is from 2012Q1 to 2012Q4 (1/1/2012 to 31/12/2012); end follow-up quarter: 2017Q4, the lead 1of 2017Q4 is 2018Q1 (1/1/2018 to 31/3/2018). However, the data of 2018 is not available, thus, the end follow-up quarter of **lag 4, lead 1 method** is 2017Q3 (lead 1 is 2017 Q4: 1/10/2017 to 31/12/2017).

(a) lag 4, lead 1 method

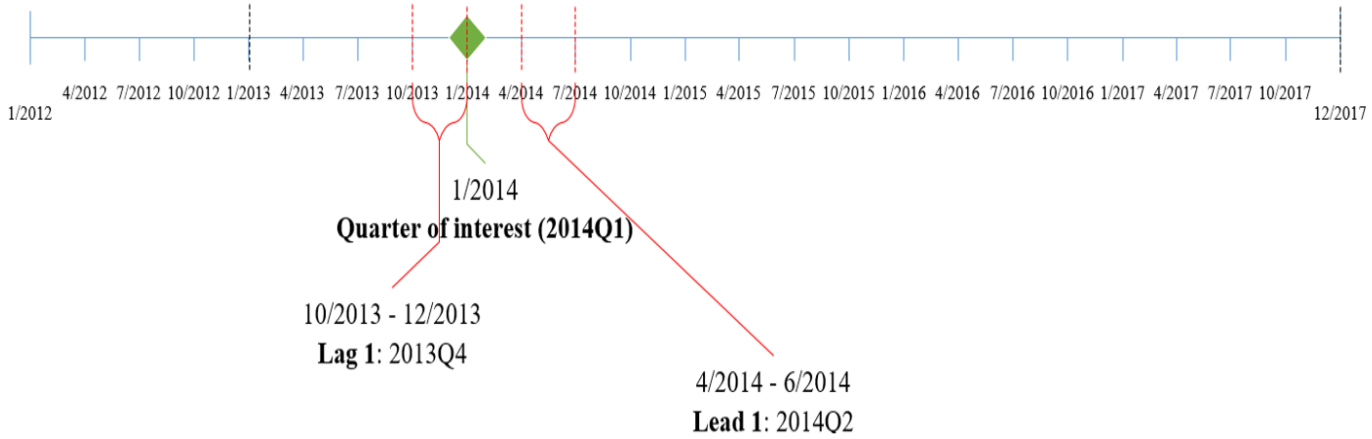

Legend: start follow-up quarter: 2013Q1, the lag 1 of 2013Q1 is 2012Q4 (1/10/2012 to 31/12/2012); end follow-up quarter: 2017Q4, the lead 1of 2017Q4 is 2018Q1 (1/1/2018 to 31/3/2018). However, the data of 2018 is not available, thus, the end follow-up quarter of **lag 1, lead 1 method** is 2017Q3 (lead 1 is 2017 Q4: 1/10/2017 to 31/12/2017).

(b) lag 1, lead 1 method

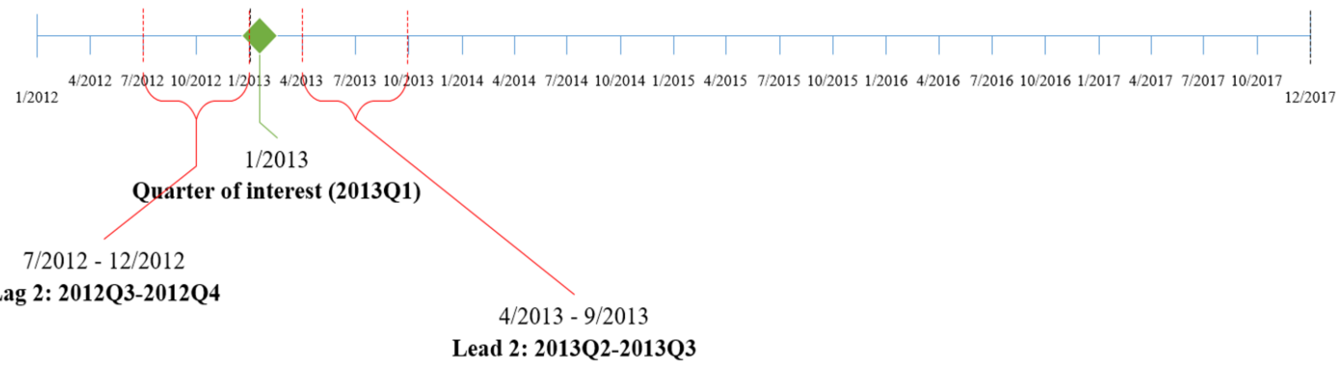

Legend: start follow-up quarter: 2013Q1, the lag 2 of 2013Q1 is from 2012Q3 to 2012Q4 (1/7/2012 to 31/12/2012); end follow-up quarter: 2017Q4, the lead 2 of 2017Q4 is from 2018Q1 to 2018Q2 (1/1/2018 to 30/6/2018). However, the data of 2018 is not available, thus, the end follow-up quarter of **lag 2, lead 2 method** is 2017Q2 (lead 2 is from 2017 Q3 to 2017Q4: 1/7/2017 to 31/12/2017).

(c) lag 2, lead 2 method

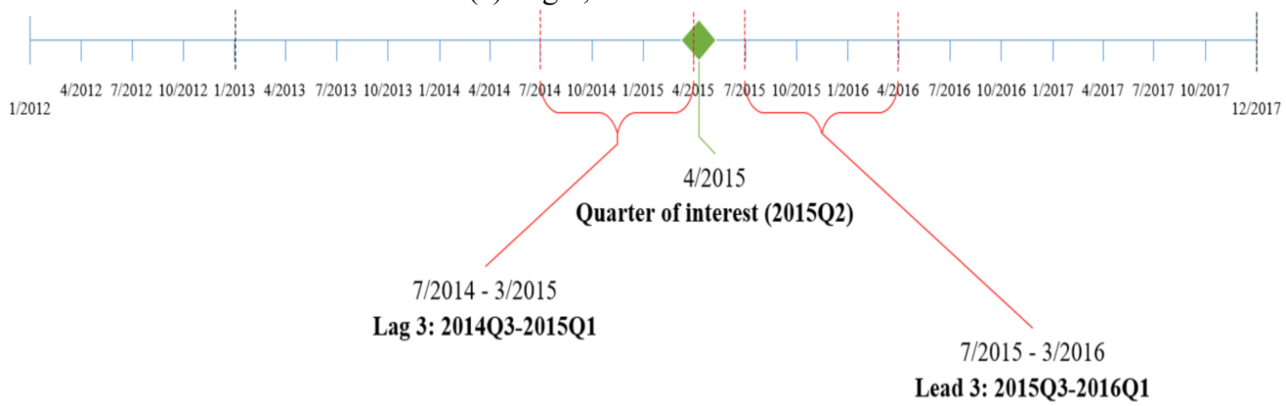

Legend: start follow-up quarter: 2013Q1, the lag 3 of 2013Q1 is from 2012Q2 to 2012Q4 (1/4/2012 to 31/12/2012); end follow-up quarter: 2017Q4, the lead 3 of 2017Q4 is from 2018Q1 to 2018Q3 (1/1/2018 to 30/9/2018). However, the data of 2018 is not available, thus, the end follow-up quarter of **lag 3, lead 3 method** is 2017Q1 (lead 3 is from 2017 Q2 to 2017Q4: 1/4/2017 to 31/12/2017).

(d) lag 3, lead 3 method

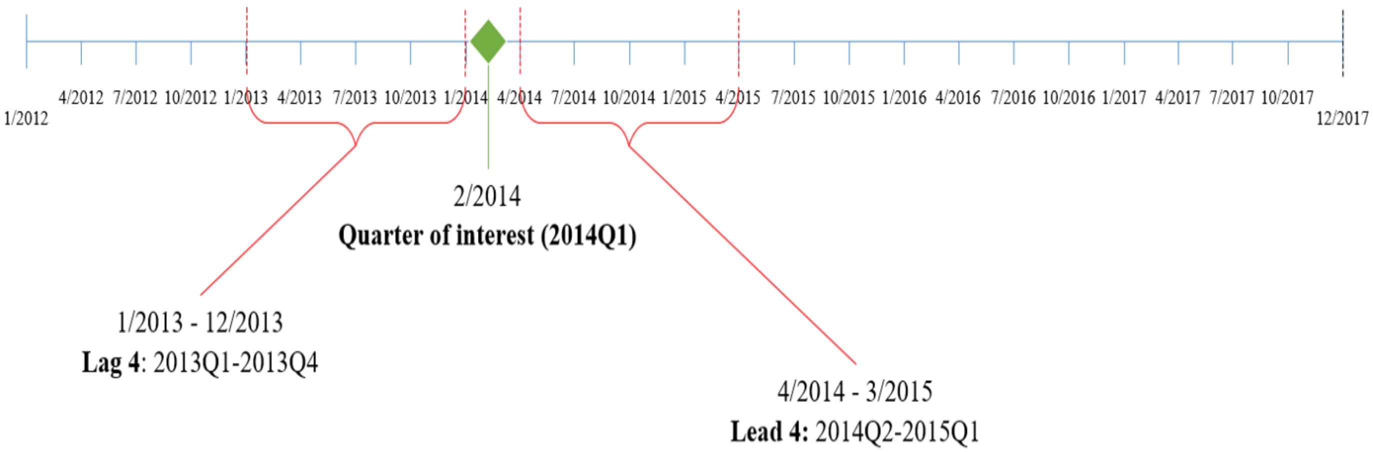

Legend: start follow-up quarter: 2013Q1, the lag 4 of 2013Q1 is from 2012Q1 to 2012Q4 (1/1/2012 to 31/12/2012); end follow-up quarter: 2017Q4, the lead 4 of 2017Q4 is from 2018Q1 to 2018Q4 (1/1/2018 to 31/12/2018). However, the data of 2018 is not available, thus, the end follow-up quarter of **lag 4, lead 4 method** is 2016Q1 (lead 4 is from 2016 Q1 to 2016Q4: 1/1/2016 to 31/12/2016).

(e) lag 4, lead 4 method
